# Metabolic reprogramming of cholesterol biosynthesis drives macrophage-mediated immune suppression in HPV-negative cervical adenocarcinoma

**DOI:** 10.3389/fimmu.2026.1808107

**Published:** 2026-05-13

**Authors:** Jianing Zhang, Wenjuan Wei, Yajun Zhang, Daqing Wang

**Affiliations:** 1 Department of Gynecology, Dalian Women and Children’s Medical Group, Dalian, Liaoning, China; 2 Central Laboratory, Dalian Women and Children’s Medical Group, Dalian, Liaoning, China; 3 Department of Anesthesiology, Dalian Women and Children’s Medical Group, Dalian, Liaoning, China; 4 Liaoning Provincial Key Laboratory, Dalian Women and Children’s Medical Group, Dalian, Liaoning, China

**Keywords:** cervical adenocarcinoma, cholesterol metabolism, DHCR7–27-HC–SPP1 axis, HPV-negative, immune microenvironment

## Abstract

**Background:**

Cervical adenocarcinoma is an increasingly common and aggressive subtype of cervical cancer with marked biological heterogeneity. Accumulating evidence suggests that HPV-positive and HPV-negative adenocarcinomas exhibit distinct immune microenvironments, but the underlying mechanisms remain unclear.

**Methods:**

Publicly available single-cell RNA sequencing datasets of cervical adenocarcinoma and normal cervical tissues were systematically analyzed using integrated bioinformatic approaches, including cell clustering, copy number variation inference, metabolic pathway analysis, and cell–cell communication modeling. Key findings were validated through *in vitro* experiments using cervical cancer cell lines, macrophage polarization assays, metabolic measurements, ELISA, immunofluorescence, and CD8^+^ T cell functional analyses.

**Results:**

Single-cell analysis revealed profound differences in cellular composition and immune states between HPV-negative and HPV-positive adenocarcinomas. HPV-negative tumors exhibited increased immune infiltration but were enriched for exhausted CD8^+^ T cells and immunosuppressive SPP1^+^ macrophages. Malignant epithelial cells from HPV-negative adenocarcinoma displayed distinct metabolic reprogramming characterized by activation of cholesterol biosynthesis pathways, elevated DHCR7 expression, and accumulation of the oxysterol 27-hydroxycholesterol (27-HC). Functionally, 27-HC induced macrophage polarization toward an immunosuppressive phenotype and promoted SPP1 secretion. Macrophage-derived SPP1, in turn, enhanced DHCR7 expression and 27-HC production in tumor cells via CD44, forming a positive feedback loop that reinforced immune suppression. Disruption of DHCR7 attenuated macrophage-mediated immunosuppression and alleviated CD8^+^ T cell exhaustion.

**Conclusions:**

This study identifies a DHCR7–27-HC–SPP1 metabolic–immune axis that drives immune escape in HPV-negative cervical adenocarcinoma, highlighting cholesterol metabolism as a potential therapeutic vulnerability.

## Graphical Abstract

**Figure:**
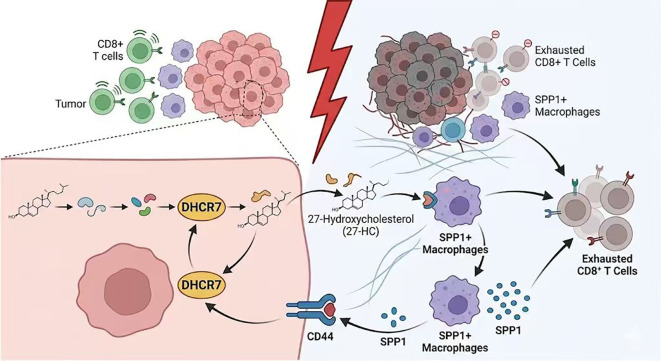

## Introduction

Cervical cancer remains a major global health burden and continues to rank among the leading causes of cancer-related mortality in women worldwide (1). Although the implementation of human papillomavirus (HPV) vaccination and cervical screening programs has significantly reduced the incidence of cervical squamous cell carcinoma, the proportion of cervical adenocarcinoma (ADC) has steadily increased over the past decades (2, 3). Compared with squamous cell carcinoma, ADC is frequently diagnosed at a more advanced stage, exhibits reduced sensitivity to radiotherapy and chemotherapy, and is associated with poorer clinical outcomes (4). Importantly, ADC represents a biologically heterogeneous disease, and growing evidence indicates that HPV-positive and HPV-negative ADCs constitute distinct molecular and clinical entities rather than variations of the same tumor type (5, 6).

Persistent infection with high-risk HPV is considered the dominant etiological factor in cervical carcinogenesis, primarily through the oncogenic activities of viral E6 and E7 proteins that disrupt p53- and RB-mediated tumor suppressive pathways (7, 8). However, a substantial subset of ADC lacks detectable HPV infection. These HPV-negative tumors display distinct genomic alterations, transcriptional programs, and epigenetic landscapes, suggesting alternative mechanisms of tumor initiation and progression (9). Clinically, HPV-negative cervical cancers are often associated with more aggressive behavior, higher recurrence rates, and inferior survival outcomes (10, 11). Moreover, accumulating clinical evidence suggests that HPV-negative cervical tumors respond poorly to immune checkpoint blockade, highlighting fundamental differences in tumor–immune interactions driven by HPV status (12). Despite these observations, the biological basis underlying the divergent immune landscapes between HPV-positive and HPV-negative ADC remains poorly understood.

The tumor immune microenvironment plays a critical role in cervical cancer development, progression, and therapeutic response (13). In HPV-positive tumors, viral antigens can elicit antiviral immune responses and promote immune cell infiltration; however, these tumors frequently evolve effective immune evasion strategies that suppress cytotoxic immunity (14). In contrast, HPV-negative cervical cancers lack viral antigens and are thought to develop within a chronically inflamed microenvironment shaped by tumor-intrinsic oncogenic and metabolic alterations (15). Bulk transcriptomic analyses have suggested differences in immune-related pathways between HPV-positive and HPV-negative cervical cancers, but these approaches are inherently limited by cellular averaging and cannot resolve the complex cellular heterogeneity within the tumor microenvironment (16).

Recent single-cell studies across multiple cancer types have demonstrated that tumor progression is accompanied by profound remodeling of immune cell composition, including T cell exhaustion, expansion of immunosuppressive myeloid populations, and functional reprogramming of stromal cells (17, 18). In cervical cancer, however, comprehensive single-cell analyses comparing HPV-positive and HPV-negative adenocarcinoma remain scarce, and the cellular mechanisms underlying immune escape in HPV-negative tumors are largely unexplored.

Among immune cell populations, T lymphocytes are central mediators of antitumor immunity. Effective tumor control relies on the presence of functional cytotoxic CD8^+^ T cells, whereas immune escape is frequently associated with T cell exhaustion characterized by sustained expression of inhibitory receptors such as PD-1, TIM-3, and LAG-3 (19). Regulatory T cells further contribute to immune suppression by limiting effector T cell activity (20). In parallel, innate immune cells—particularly tumor-associated macrophages—play a pivotal role in shaping the immunological tone of the tumor microenvironment (21). Macrophages exhibit remarkable plasticity and can adopt either pro-inflammatory or immunosuppressive phenotypes in response to local cues. Increasing evidence indicates that SPP1-expressing macrophages represent a distinct immunosuppressive subset that promotes T cell exhaustion, facilitates tumor invasion, and correlates with poor prognosis across multiple cancer types (22). Whether such macrophage programs are preferentially enriched in HPV-negative ADC and how they are regulated remain unknown.

In this study, we leveraged publicly single-cell RNA sequencing datasets of cervical adenocarcinoma to comprehensively dissect the cellular composition and immune landscape associated with HPV status. Through integrative single-cell, metabolic, and cell–cell communication analyses, we identified a DHCR7-driven cholesterol metabolic program that links tumor-intrinsic metabolic reprogramming to macrophage-mediated immune suppression in HPV-negative adenocarcinoma. These findings provide mechanistic insight into the distinct tumor microenvironment of HPV-negative cervical adenocarcinoma and suggest metabolism-based therapeutic vulnerabilities for this aggressive disease subtype.

## Methods

### Single-cell RNA sequencing data analysis

Publicly available single-cell RNA sequencing (scRNA-seq) data of ADC were obtained from the Gene Expression Omnibus (GEO) database. Specifically, dataset GSE197461 was used to include five ADC samples, comprising three HPV-positive adenocarcinomas and two HPV-negative adenocarcinomas, while two normal cervical tissue samples were obtained from dataset GSE208653 and used as controls. Raw gene expression matrices and corresponding metadata were downloaded and processed using the Seurat package in R. Cells with fewer than 200 detected genes, more than 6,000 detected genes, or mitochondrial gene content exceeding 20% were excluded, and genes expressed in fewer than three cells were removed. After quality control, data were normalized using the LogNormalize method, highly variable genes were identified, and principal component analysis was performed. To correct for batch effects across datasets (GSE197461 and GSE208653), we applied Canonical Correlation Analysis (CCA) using the Seurat v4 integration workflow. Briefly, after log-normalization and identification of highly variable genes, the FindIntegrationAnchors function was used with dims = 1:30 to identify pairwise correspondences between cells across datasets. These anchors were then used to harmonize the datasets via the IntegrateData function, generating a batch-corrected expression matrix for downstream analysis. Principal component analysis (PCA) was performed on the integrated data, and the first 50 principal components were selected for downstream analyses based on elbow plot inspection. Graph-based clustering was performed using the FindNeighbors and FindClusters functions (resolution = 0.5), and dimensionality reduction for visualization was achieved using Uniform Manifold Approximation and Projection (UMAP). The effectiveness of batch correction was assessed by visualizing the distribution of cells from different samples in the UMAP plots, which showed substantial mixing of cells across batches, indicating successful data harmonization. Cell types were annotated based on canonical marker genes. Copy number variation (CNV) profiles were inferred using the inferCNV package, with normal epithelial cells serving as reference to distinguish malignant epithelial populations and to compare genomic instability between HPV-positive and HPV-negative adenocarcinomas. Single-cell metabolic pathway activities were quantified using the scMetabolism package based on KEGG pathway gene sets, and pathway scores were calculated at the single-cell level to assess metabolic reprogramming across different cell types and HPV status groups. In addition, transcription factor regulatory activity was analyzed using the SCENIC workflow to infer regulon activity. Intercellular communication networks were inferred using the CellChat package by evaluating ligand–receptor interactions among annotated cell populations, and differential signaling pathways between HPV-positive and HPV-negative adenocarcinomas were identified. All statistical analyses were performed in R, and adjusted P values < 0.05 were considered statistically significant.

### Cell culture and treatment

Human ADC cell lines C33A (HPV-negative), HeLa (HPV-positive), and SiHa (HPV-positive), as well as the human monocytic cell line THP-1, were obtained from the Cell Bank of the Chinese Academy of Sciences. C33A, HeLa, and SiHa cells were cultured in Dulbecco’s Modified Eagle Medium (DMEM) supplemented with 10% fetal bovine serum (FBS), 100 U/mL penicillin, and 100 μg/mL streptomycin. THP-1 cells were maintained in RPMI-1640 medium containing 10% FBS and antibiotics. All cells were cultured at 37 °C in a humidified incubator with 5% CO_2_.

Peripheral blood mononuclear cells (PBMCs) were isolated from peripheral blood of healthy donors using density gradient centrifugation. CD8^+^ T cells were purified using a magnetic bead-based CD8^+^ T cell isolation kit (Miltenyi Biotec). Purified CD8^+^ T cells were resuspended in RPMI-1640 medium supplemented with 10% FBS and 100 U/mL IL-2 and activated with anti-CD3 (1 μg/mL) and anti-CD28 (1 μg/mL) antibodies for 24 h prior to downstream experiments.

To generate macrophages, THP-1 cells were treated with phorbol 12-myristate 13-acetate (PMA, 100 ng/mL) for 24 h, followed by replacement with fresh medium before subsequent treatments.

### Cell transfection

Small interfering RNAs (siRNAs) targeting DHCR7 (si-DHCR7#1 and si-DHCR7#2), CD44 (si-CD44#1 and si-CD44#2), and a non-targeting negative control siRNA (si-NC) were purchased from GenePharma (Shanghai, China). Cells were seeded in 6-well plates and transfected at 60–70% confluence using Lipofectamine 3000 (Invitrogen) according to the manufacturer’s instructions. After 48 h of transfection, knockdown efficiency was validated by quantitative real-time PCR, and the siRNA with the highest silencing efficiency was selected for subsequent experiments.

### Preparation of tumor cell–conditioned medium

C33A cells transfected with si-NC or si-DHCR7#2 were cultured for 48 h, after which the medium was replaced with serum-free DMEM and cells were incubated for an additional 24 h. Cell culture supernatants were then collected, centrifuged, and filtered through 0.22 μm filters to remove cell debris. The resulting tumor cell–conditioned medium (TCM) was stored at −20 °C until use.

### Quantitative real-time PCR

Total RNA was extracted from cells using TRIzol reagent (Invitrogen) according to the manufacturer’s protocol. Complementary DNA (cDNA) was synthesized using the PrimeScript RT Reagent Kit (TaKaRa). Quantitative real-time PCR was performed using SYBR Premix Ex Taq II (TaKaRa) on a real-time PCR system. The amplification conditions were as follows: initial denaturation at 95 °C for 30 s, followed by 40 cycles of 95 °C for 5 s and 60 °C for 30 s. Melting curve analysis was conducted to confirm amplification specificity. GAPDH was used as an internal control, and relative gene expression levels were calculated using the 2^-^ΔΔCt method. Primer sequences are listed in the Supplementary Materials.

### Quantification of cholesterol metabolites

For intracellular metabolite analysis, cells were harvested and lysed with pre-chilled methanol, followed by vortexing and centrifugation at 12,000 rpm for 15 min at 4 °C. The supernatants were subjected to high-performance liquid chromatography–tandem mass spectrometry (HPLC–MS/MS) to quantify 27-hydroxycholesterol (27-HC). Chromatographic separation was performed using a C18 column, and detection was carried out in positive electrospray ionization mode using selected reaction monitoring (SRM).

For extracellular 27-HC measurement, culture supernatants from C33A cells were collected and analyzed using a commercial human 27-hydroxycholesterol ELISA kit according to the manufacturer’s instructions.

### Enzyme-linked immunosorbent assay

The concentration of secreted SPP1 in macrophage culture supernatants was measured using a human SPP1 (osteopontin) ELISA kit following the manufacturer’s protocol. Briefly, standard solutions and samples were added to 96-well plates pre-coated with capture antibodies and incubated at 37 °C for 2 h. After washing to remove unbound components, a biotinylated detection antibody was added and incubated at 37 °C for 1 h. Subsequently, horseradish peroxidase (HRP)-conjugated secondary antibody was added and incubated at 37 °C for 30 min. After thorough washing, tetramethylbenzidine (TMB) substrate was added for color development, and the reaction was terminated by adding stop solution. Absorbance was measured at 450 nm using a microplate reader, and SPP1 concentrations were calculated based on standard curves.

### Cell viability assay

Cell viability was evaluated using the Cell Counting Kit-8 (CCK-8, Beyotime). C33A cells were seeded in 96-well plates at a density of 1 × 10^4^ cells per well and cultured for 24 h. After the indicated treatments, 10 μL of CCK-8 reagent was added to each well and incubated for 2 h at 37 °C. Absorbance at 450 nm was measured using a microplate reader. All experiments were performed in triplicate.

### Immunofluorescence staining

For immunofluorescence, cells cultured on coverslips were fixed with 4% paraformaldehyde for 30 minutes, permeabilized using 0.1% Triton X-100 for 15 minutes, and then blocked with 5% bovine serum albumin for 1 hour. Primary antibodies against CD206 or ARG1 were applied and incubated overnight at 4 °C. After washing, samples were incubated with fluorescence-conjugated secondary antibodies for 1 hour at room temperature. Nuclei were visualized with DAPI staining. Images were acquired using a fluorescence microscope, and mean fluorescence intensity was quantified with ImageJ software.

### Flow cytometry analysis

CD8^+^ T cells treated with different conditioned media were collected, washed, and stained with fluorochrome-conjugated antibodies against PD-1 and TIM-3 at 4 °C for 30 min in the dark. After washing, cells were analyzed using a BD FACSCanto II flow cytometer, and data were processed using FlowJo software. For apoptosis analysis, cells were stained with Annexin V-FITC and propidium iodide according to the manufacturer’s instructions and analyzed by flow cytometry.

### Statistical analysis

All experiments were performed in at least three independent replicates. Data are presented as mean ± standard deviation (SD). Statistical comparisons between two groups were conducted using the Student’s t-test, while differences among multiple groups were analyzed by one-way analysis of variance (ANOVA). Analyses were carried out with GraphPad Prism 9.0 software, and a P-value < 0.05 was considered statistically significant.

## Results

### Single-cell landscape reveals distinct cellular compositions between HPV-positive and HPV-negative ADC

**Figure 1:**
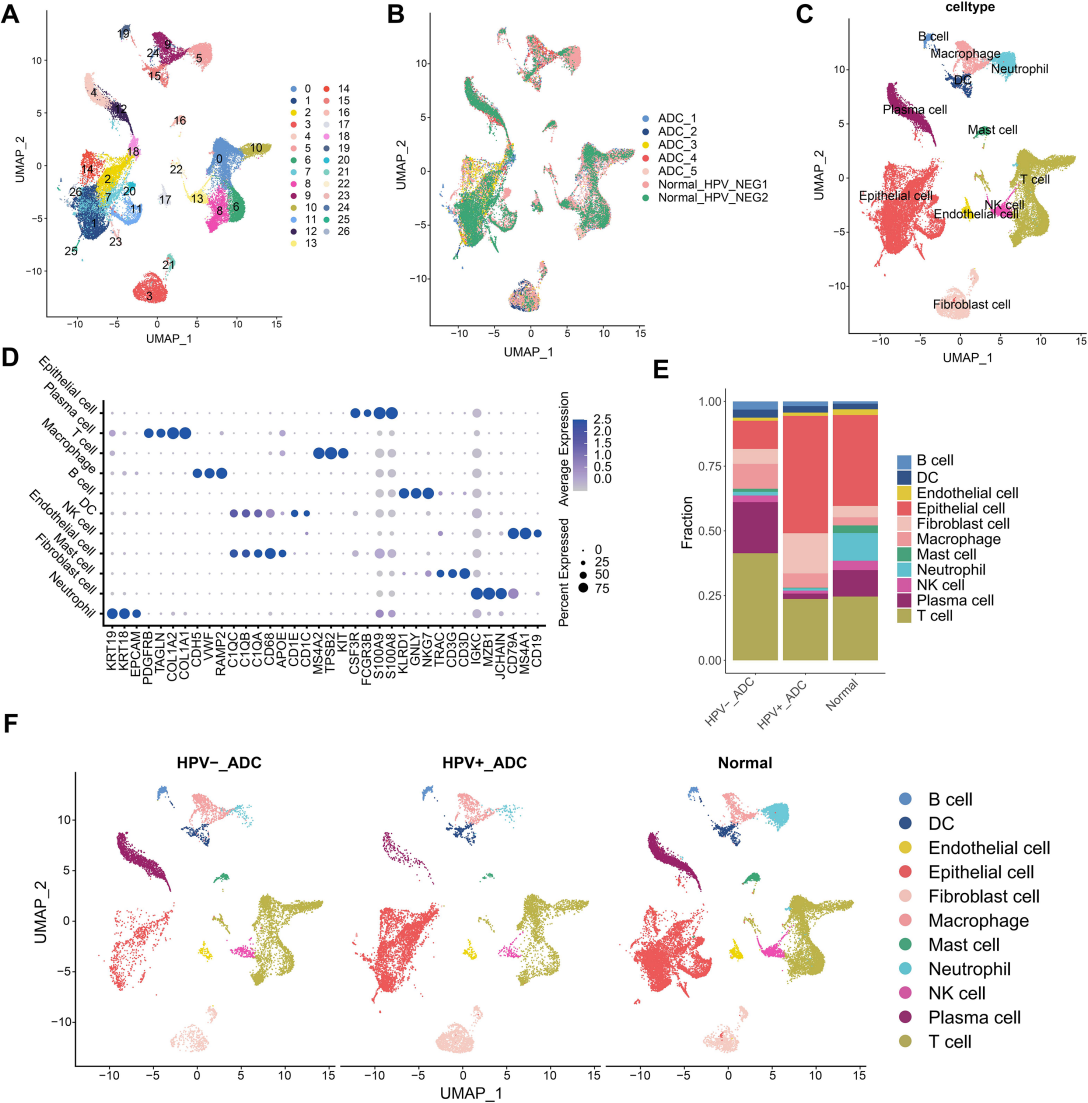
Single-cell transcriptomic landscape of HPV-positive and HPV-negative cervical adenocarcinoma. **(A)** Uniform manifold approximation and projection (UMAP) visualization of all cells after batch correction, identifying 27 distinct cell clusters across cervical adenocarcinoma and normal cervical tissues. **(B)** UMAP plot showing the distribution of cells from different samples, including HPV-positive adenocarcinoma, HPV-negative adenocarcinoma, and normal controls. **(C)** UMAP visualization of major cell types annotated based on canonical marker gene expression. **(D)** Dot plot showing the expression of representative marker genes used for annotation of each major cell population. **(E)** Bar plot depicting the relative proportions of major cell types across different sample groups. **(F)** UMAP visualization highlighting the spatial distribution of different sample groups across the cellular landscape.

Single-cell transcriptomic profiling was performed on ADC and normal cervical tissues, followed by batch correction using CCA. After dimensionality reduction and unsupervised clustering, a total of 27 distinct cell clusters were identified and visualized by UMAP (**Figures 1A, B**). Based on the expression of established canonical marker genes, these clusters were annotated into 11 major cell populations, including lymphoid cells (B cells, plasma cells, T cells, and NK cells), myeloid cells (macrophages, neutrophils, dendritic cells, and mast cells), and stromal cells (endothelial cells, epithelial cells, and fibroblasts) (**Figures 1C, D**).

Comparative analysis demonstrated that, relative to normal controls, ADC exhibited a pronounced reduction in neutrophil abundance, accompanied by a significant expansion of fibroblasts and macrophages (**Figure 1E**). Furthermore, stratification by HPV status revealed distinct immune and stromal profiles between HPV-negative and HPV-positive adenocarcinomas. HPV-negative adenocarcinomas displayed a higher proportion of immune cells, particularly lymphocytes, whereas stromal components, including epithelial cells and cancer-associated fibroblasts (CAFs), were relatively reduced compared with HPV-positive tumors (**Figure 1F**). In contrast, HPV-positive adenocarcinomas were characterized by lower overall immune cell infiltration, indicating a more immune-excluded tumor microenvironment. Collectively, these findings indicate that HPV-negative and HPV-positive ADCs are associated with fundamentally different tumor microenvironment architectures, suggesting divergent immune regulatory states and potentially distinct oncogenic mechanisms.

### Immune cell heterogeneity highlights distinct humoral and cellular immune states in ADC

**Figure 2:**
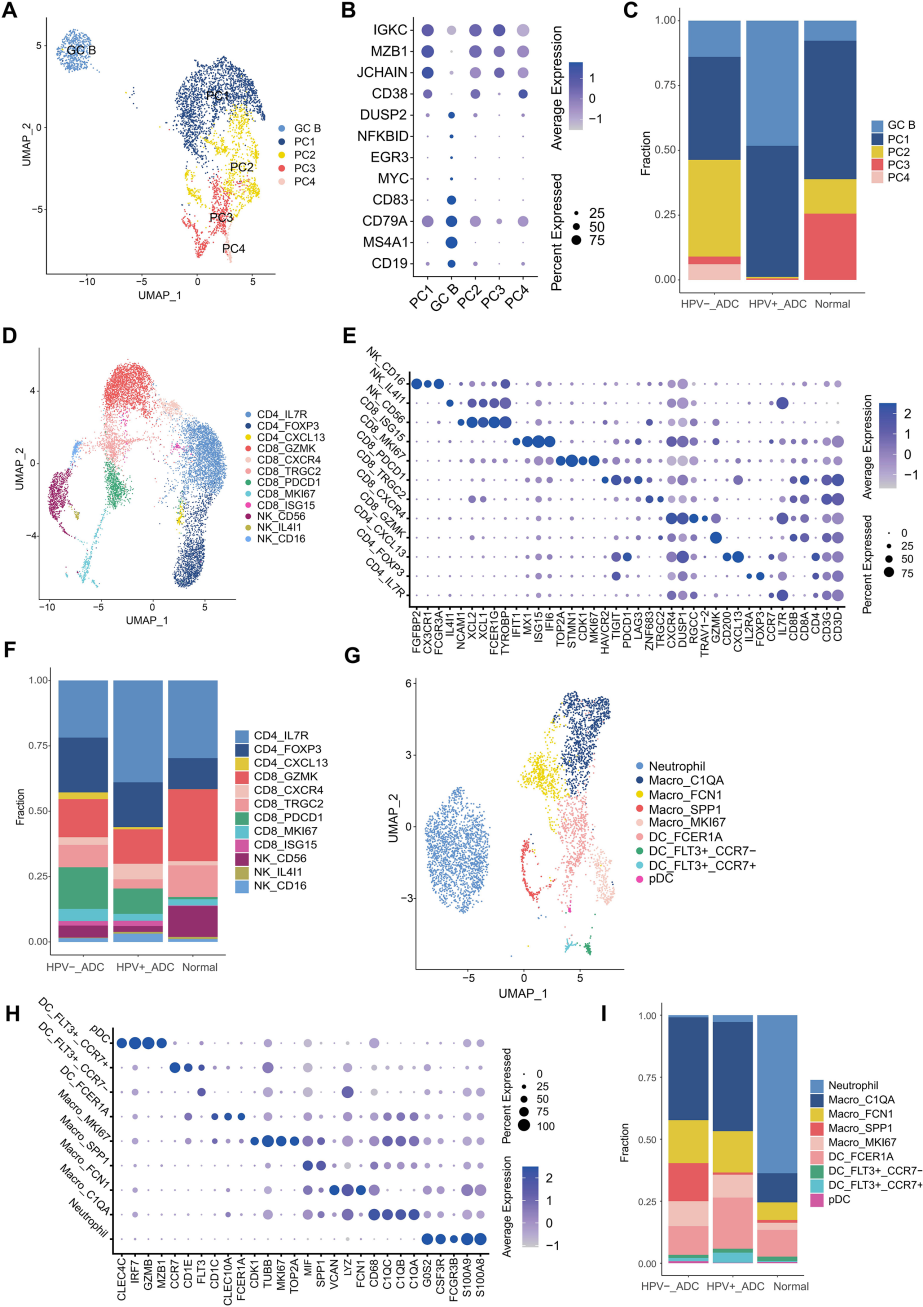
Immune cell heterogeneity in cervical adenocarcinoma. **(A)** UMAP visualization of B and plasma cell subclusters. **(B)** Dot plot showing canonical marker genes used to annotate B/plasma cell subpopulations. **(C)** Relative proportions of B/plasma cell subpopulations across normal tissues, HPV-positive adenocarcinoma, and HPV-negative adenocarcinoma. **(D)** UMAP visualization of T and NK cell subclusters. **(E)** Dot plot displaying marker gene expression used for annotation of T/NK cell subpopulations. **(F)** Relative proportions of T/NK cell subpopulations across different sample groups. **(G)** UMAP visualization of myeloid cell subclusters. **(H)** Dot plot showing canonical markers for annotation of myeloid cell subpopulations. **(I)** Relative proportions of myeloid cell subpopulations across normal tissues, HPV-positive adenocarcinoma, and HPV-negative adenocarcinoma.

Subclustering of the B/plasma cell compartment identified five distinct subpopulations (PC1–PC5) with clear separation on UMAP (**Figures 2A, B**). These subsets exhibited pronounced intergroup heterogeneity across normal tissues, HPV-positive adenocarcinoma, and HPV-negative adenocarcinoma. PC2 was nearly absent in HPV-positive adenocarcinoma, whereas PC4 was predominantly derived from HPV-negative adenocarcinoma, and PC3 mainly originated from normal tissues. Functionally, PC1 was characterized by high immunoglobulin expression and enrichment of endoplasmic reticulum stress–related pathways, consistent with a highly secretory plasma cell state. In contrast, PC2 exhibited transcriptional features suggestive of germinal center–derived plasma cells and was enriched for humoral immune response pathways, indicating a sustained adaptive immune response in HPV-negative tumors (**Figure 2C**).

Re-clustering of T and NK cells identified 12 distinct subpopulations (**Figures 2D, E**). Compared with normal tissues, both HPV-positive and HPV-negative adenocarcinomas showed a marked reduction in cytotoxic CD8^+^ T cells (CD8_GZMK), accompanied by an increased proportion of exhausted CD8^+^ T cells (CD8_PDCD1) and regulatory T cells, indicating T cell dysfunction and immune evasion. In addition, CD56^+^ NK cells were significantly reduced in adenocarcinoma samples relative to normal controls (**Figure 2F**).

Subclustering of myeloid cells revealed nine distinct subpopulations with substantial heterogeneity between normal and tumor samples (**Figures 2G, H**). Neutrophils were almost exclusively derived from normal tissues. Notably, SPP1^+^ macrophages were predominantly enriched in HPV-negative adenocarcinoma, whereas FCER1A^+^ dendritic cells were increased in HPV-positive adenocarcinoma (**Figure 2I**), highlighting divergent myeloid remodeling patterns associated with HPV status.

### Cancer-associated fibroblast and epithelial heterogeneity defines distinct malignant states in ADC

Subclustering analysis of cancer-associated fibroblasts (CAFs) identified eight distinct CAF subpopulations (**Supplementary Figures 1A, B**). Comparative analysis revealed marked differences in CAF composition according to HPV status. Specifically, CCL2^+^ CAFs were significantly reduced in HPV-positive adenocarcinoma, whereas CYP1B1^+^ CAFs were markedly enriched in HPV-positive tumors (**Supplementary Figure 1C**), indicating distinct stromal remodeling patterns associated with HPV infection. In parallel, epithelial cells were further subclustered to resolve malignant and non-malignant populations (**Supplementary Figure 2A**). InferCNV analysis demonstrated clear copy number variation (CNV) differences between normal epithelial cells and tumor cells (**Supplementary Figure 2B**), confirming malignant identity. Notably, tumor cells from HPV-negative adenocarcinoma exhibited overall lower CNV levels compared with HPV-positive tumors (**Supplementary Figure 2C**), suggesting distinct genomic instability states.

Gene program scoring further revealed functional heterogeneity among malignant epithelial cells. Stress-related and mesenchymal gene signatures were preferentially enriched in specific malignant subpopulations, indicating divergent cellular states associated with stress adaptation and epithelial–mesenchymal transition (**Supplementary Figures 3A, B**). Gene set variation analysis (GSVA) using hallmark pathways demonstrated substantial metabolic, immune, and proliferative differences among malignant subclusters (**Supplementary Figure 3C**). KEGG pathway enrichment analysis further classified malignant cells into five functional groups: ADC_1 and ADC_3 were enriched for cell cycle–related pathways, including cell cycle progression and DNA replication; ADC_2 was associated with N-glycan biosynthesis; ADC_4 exhibited enhanced energy metabolism, including carbon metabolism, oxidative phosphorylation, thermogenesis, and glycolysis/gluconeogenesis; and ADC_5 was enriched for immune-related pathways such as antigen processing and presentation, endocytosis, NOD-like receptor signaling, and Th17 cell differentiation (**Supplementary Figure 3D**).

### Steroid metabolic reprogramming characterizes malignant cells in HPV-negative ADC

**Figure 3:**
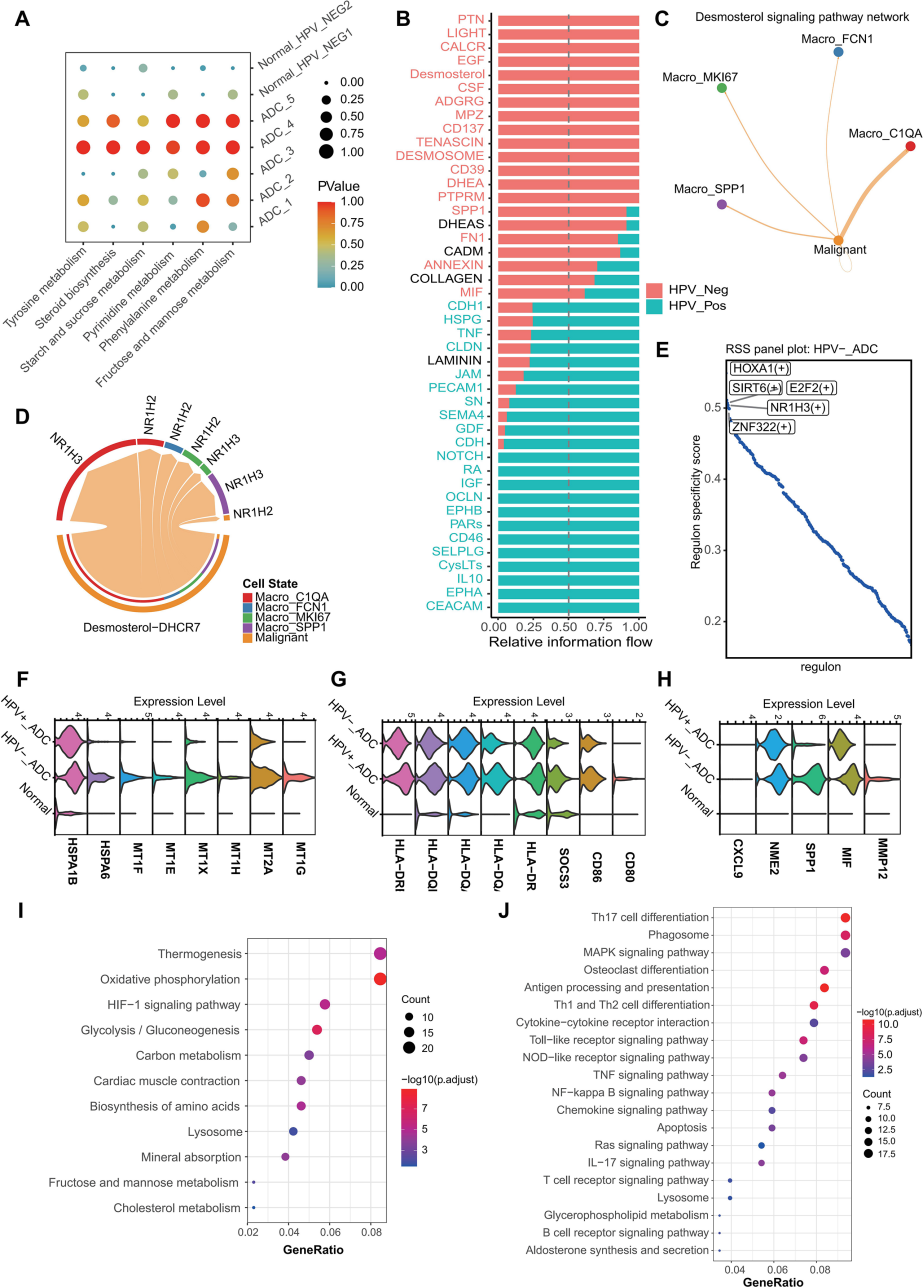
Steroid metabolic reprogramming and macrophage interaction in cervical adenocarcinoma. **(A)** Metabolic pathway activity analysis of epithelial cells across different sample groups. **(B)** Differential expression of steroid-related metabolites and genes between HPV-negative and HPV-positive adenocarcinoma. **(C)** Cell–cell communication analysis between malignant epithelial cells and macrophages. **(D)** Predicted interaction between tumor cells and macrophages mediated through the DHCR7–NR1H3 axis. **(E)** SCENIC analysis showing transcription factor regulon activity across malignant epithelial cells, highlighting NR1H3. **(F–H)** Expression levels of metallothionein genes, heat shock protein genes, and inflammatory cytokines in Macro_C1QA cells across different sample groups. **(I)** KEGG pathway enrichment analysis of genes upregulated in Macro_C1QA cells. **(J)** KEGG pathway enrichment analysis of genes downregulated in Macro_C1QA cells.

To identify potential metabolic regulators that might influence the immune microenvironment, we integrated two complementary analytical approaches. First, cell-cell communication analysis using CellChat revealed that interactions between malignant cells and macrophages were markedly enriched in HPV-negative tumors compared to HPV-positive tumors (**Figure 3A**). Among the top differentially active ligand-receptor pairs, the SPP1–CD44 axis was specifically upregulated in HPV-negative adenocarcinoma (**Figure 3B**), consistent with our earlier finding that SPP1+ macrophages are selectively enriched in this subtype (**Figure 2I**). Parallel to this, we performed metabolic pathway analysis on malignant epithelial cells, which revealed that the steroid biosynthesis pathway was specifically upregulated in HPV-negative ADC (**Figure 3C**). Within this pathway, the cholesterol intermediate desmosterol was selectively enriched in HPV-negative malignant cells (**Figure 3D**). Accordingly, expression of DHCR7, the enzyme catalyzing desmosterol-to-cholesterol conversion, and its downstream nuclear receptor NR1H3 (LXRα) were both significantly increased in HPV-negative tumors (**Figures 3E, F**). SCENIC analysis further identified NR1H3 as a top-ranked regulon specifically activated in HPV-negative ADC (**Figure 3G**), indicating a transcriptional program linking cholesterol metabolism to tumor-specific regulatory networks. The convergence of these two analytical lines—SPP1-CD44-mediated macrophage interactions and DHCR7-driven cholesterol metabolism—led us to hypothesize a functional link between tumor metabolic reprogramming and macrophage-mediated immunosuppression in HPV-negative ADC. Specifically, we proposed that DHCR7-derived metabolites might influence macrophage polarization, while macrophage-derived SPP1 might in turn regulate DHCR7 expression in tumor cells, forming a bidirectional feedback loop. This hypothesis guided our subsequent experimental validation.

### Metabolic reprogramming of malignant cells shapes immunosuppressive macrophage states in HPV-negative adenocarcinoma

Given the metabolic alterations observed in malignant cells, we next explored their impact on macrophage function. Functional comparison of the Macro_C1QA population revealed profound differences between HPV-positive and HPV-negative adenocarcinoma. In HPV-negative tumors, activation of the DHCR7–NR1H3 axis was associated with upregulation of metallothionein and heat shock protein–related genes in Macro_C1QA cells, indicating an oxidative stress–responsive state, accompanied by increased expression of anti-inflammatory factors (**Figures 3F–H**). Gene enrichment analysis showed that upregulated genes in Macro_C1QA were significantly associated with cholesterol metabolism pathways (**Figure 3I**), whereas multiple inflammation-related pathways were suppressed (**Figure 3J**).

**Figure 4:**
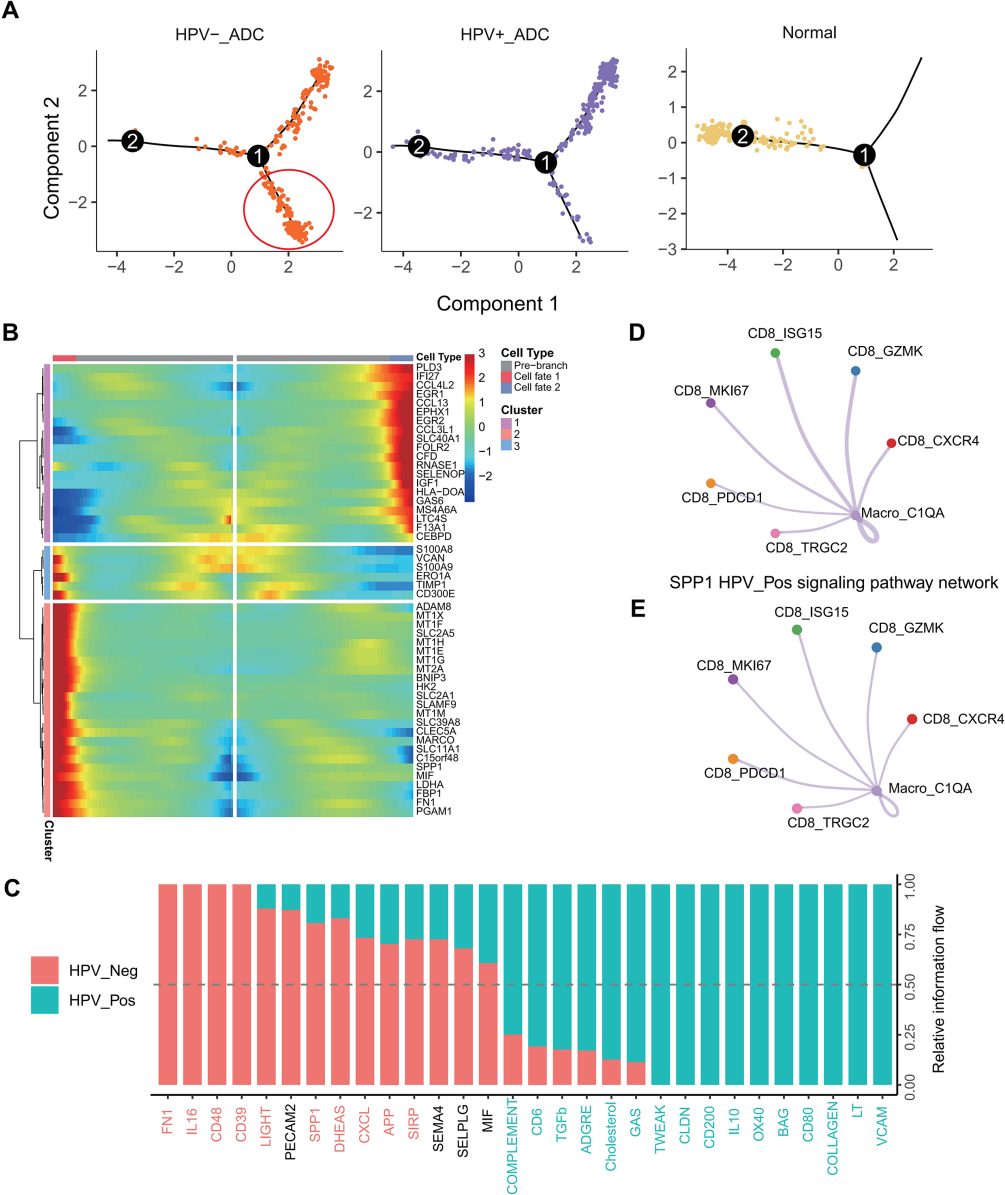
Pseudotime trajectory and cell–cell communication of Macro_C1QA cells **(A)** Pseudotime trajectory analysis revealing a distinct evolutionary path of Macro_C1QA cells in HPV-negative cervical adenocarcinoma. **(B)** Dynamic expression patterns of immunosuppressive and stress-related genes, including *SPP1*, *MIF*, and metallothioneins, along the pseudotime trajectory. **(C–E)** Cell–cell communication analysis showing that metabolically reprogrammed Macro_C1QA cells predominantly interact with CD8^+^ T cells through the SPP1–CD44 ligand–receptor axis in HPV-negative tumors.

Pseudotime trajectory analysis further demonstrated a distinct evolutionary trajectory of Macro_C1QA cells in HPV-negative adenocarcinoma (**Figure 4A**). Along this trajectory, macrophages progressively upregulated immunosuppressive and stress-related genes, including SPP1, MIF, and metallothioneins (**Figure 4B**). Finally, cell–cell communication analysis revealed that in HPV-negative tumors, metabolically reprogrammed Macro_C1QA cells interacted with CD8^+^ T cells predominantly through the SPP1–CD44 ligand–receptor axis, thereby contributing to suppression of CD8^+^ T cell immune responses (**Figures 4C–E**).

### DHCR7 is highly expressed in HPV-negative ADC cells and promotes accumulation of the cholesterol intermediate 27-hydroxycholesterol

**Figure 5:**
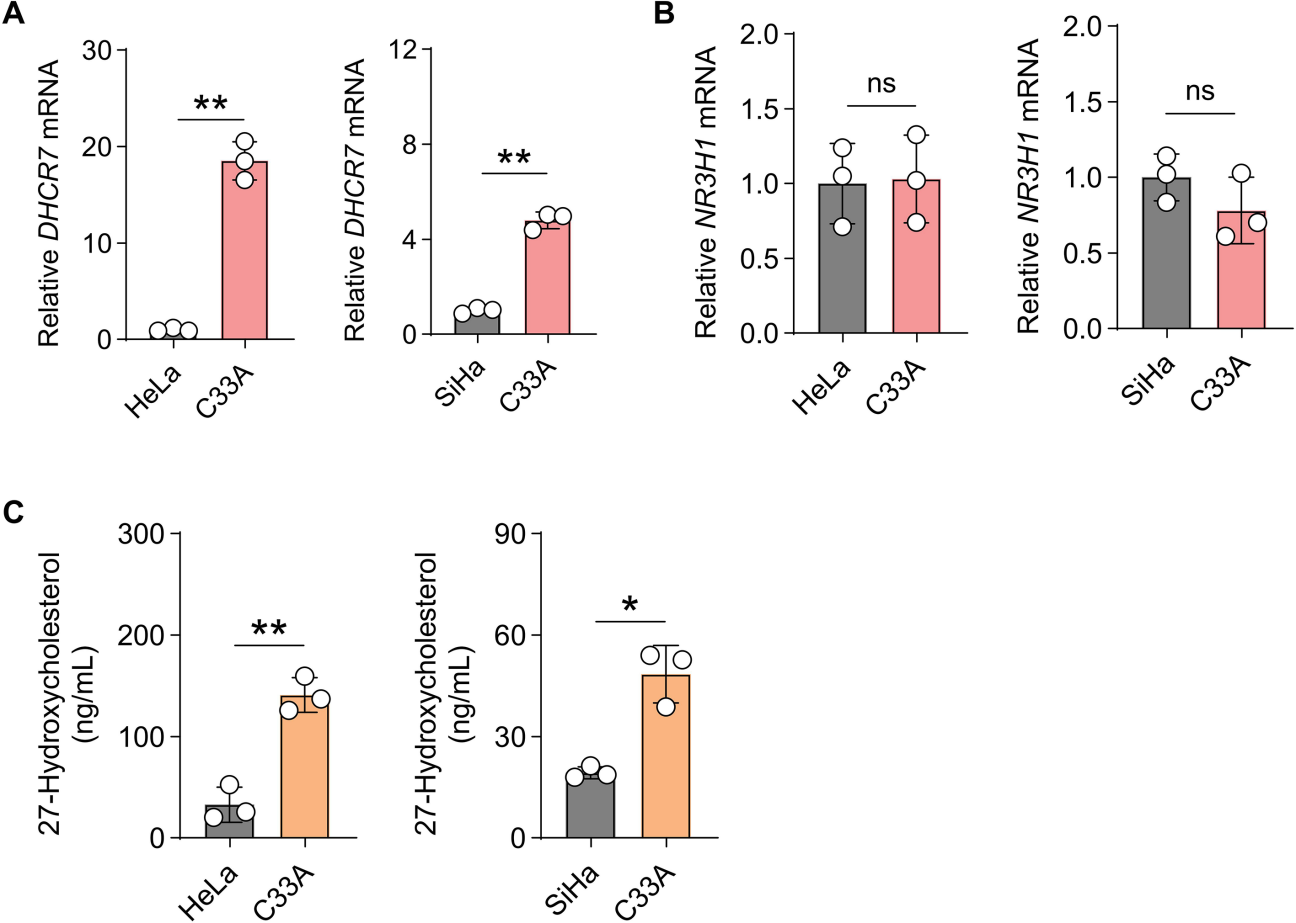
DHCR7 is highly expressed in HPV-negative cervical adenocarcinoma cells and promotes 27-hydroxycholesterol accumulation. **(A)** Quantitative real-time PCR (qPCR) analysis of *DHCR7* mRNA expression in HPV-negative C33A cells and HPV-positive HeLa and SiHa cells. **(B)** qPCR analysis of *NR3H1* mRNA expression in C33A, HeLa, and SiHa cells. **(C)** Quantification of intracellular 27-hydroxycholesterol (27-HC) levels in C33A, HeLa, and SiHa cells. Data are presented as mean ± SD. ns, not significant. * p < 0.05. ** p < 0.01.

We first examined the transcriptional expression of DHCR7 in HPV-negative ADC cells (C33A) and HPV-positive cervical cancer cell lines (HeLa and SiHa). Quantitative real-time PCR analysis demonstrated that DHCR7 mRNA expression was significantly upregulated in C33A cells compared with HeLa and SiHa cells (**Figure 5A**). In contrast, the expression of the lipid metabolism–related regulatory factor NR3H1 showed no significant difference between HPV-negative and HPV-positive cell lines (**Figure 5B**), suggesting that elevated DHCR7 expression represents a distinct metabolic feature of HPV-negative ADC cells. Consistently, metabolite quantification revealed that intracellular levels of 27-hydroxycholesterol (27-HC), a key intermediate of the cholesterol biosynthesis pathway, were markedly higher in C33A cells than in HeLa and SiHa cells (**Figure 5C**). Together, these results indicate that DHCR7 is selectively upregulated in HPV-negative ADC cells and is associated with aberrant cholesterol metabolism and 27-HC accumulation.

### 27-HC dose-dependently induces immunosuppressive macrophage polarization and promotes SPP1 secretion

**Figure 6:**
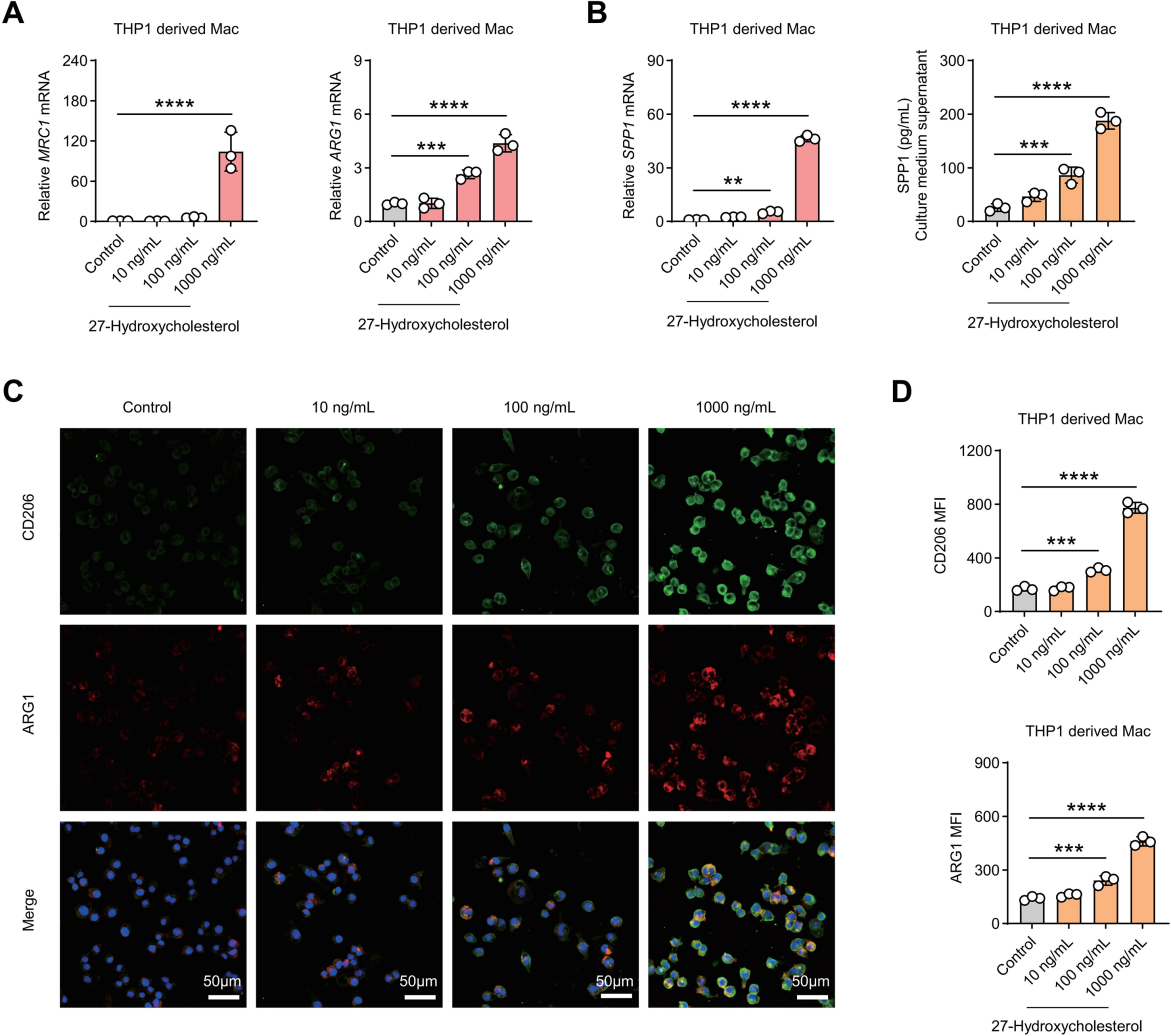
27-hydroxycholesterol induces immunosuppressive macrophage polarization and promotes SPP1 secretion. **(A)** qPCR analysis of *MRC1* (CD206), *ARG1*, and *SPP1* mRNA expression in THP-1–derived macrophages treated with increasing concentrations of 27-HC (0, 10, 100, 1000 ng/mL). **(B)** ELISA quantification of SPP1 secretion in macrophage culture supernatants following 27-HC treatment. **(C, D)** Immunofluorescence staining showing CD206 (green) and ARG1 (red) expression in macrophages after 27-HC treatment. Nuclei were counterstained with DAPI (blue). Scale bar, 50 μm. ** p < 0.01, *** p < 0.001, **** p < 0.0001.

Given the abnormal accumulation of 27-HC in C33A cells, we next investigated its effects on macrophage polarization. THP-1 cells were differentiated into macrophages using PMA and subsequently treated with increasing concentrations of 27-HC (10, 100, and 1000 ng/mL). qPCR analysis revealed that expression levels of M2-associated immunosuppressive markers CD206 and ARG1 increased in a dose-dependent manner following 27-HC treatment. Notably, transcription of the immunoregulatory factor SPP1 was also significantly upregulated in response to increasing 27-HC concentrations (**Figure 6A**). ELISA further confirmed that SPP1 secretion in macrophage culture supernatants increased progressively with higher doses of 27-HC (**Figure 6B**). Immunofluorescence staining showed markedly enhanced protein expression of CD206 and ARG1 in macrophages following 27-HC stimulation (**Figures 6C, D**). These findings demonstrate that 27-HC can directly drive macrophage polarization toward an M2-like immunosuppressive phenotype and promote SPP1 secretion, consistent with the chronic inflammatory yet immunosuppressed tumor microenvironment observed in HPV-negative ADC.

### DHCR7 knockdown reverses C33A-induced macrophage M2 polarization and SPP1 secretion

**Figure 7:**
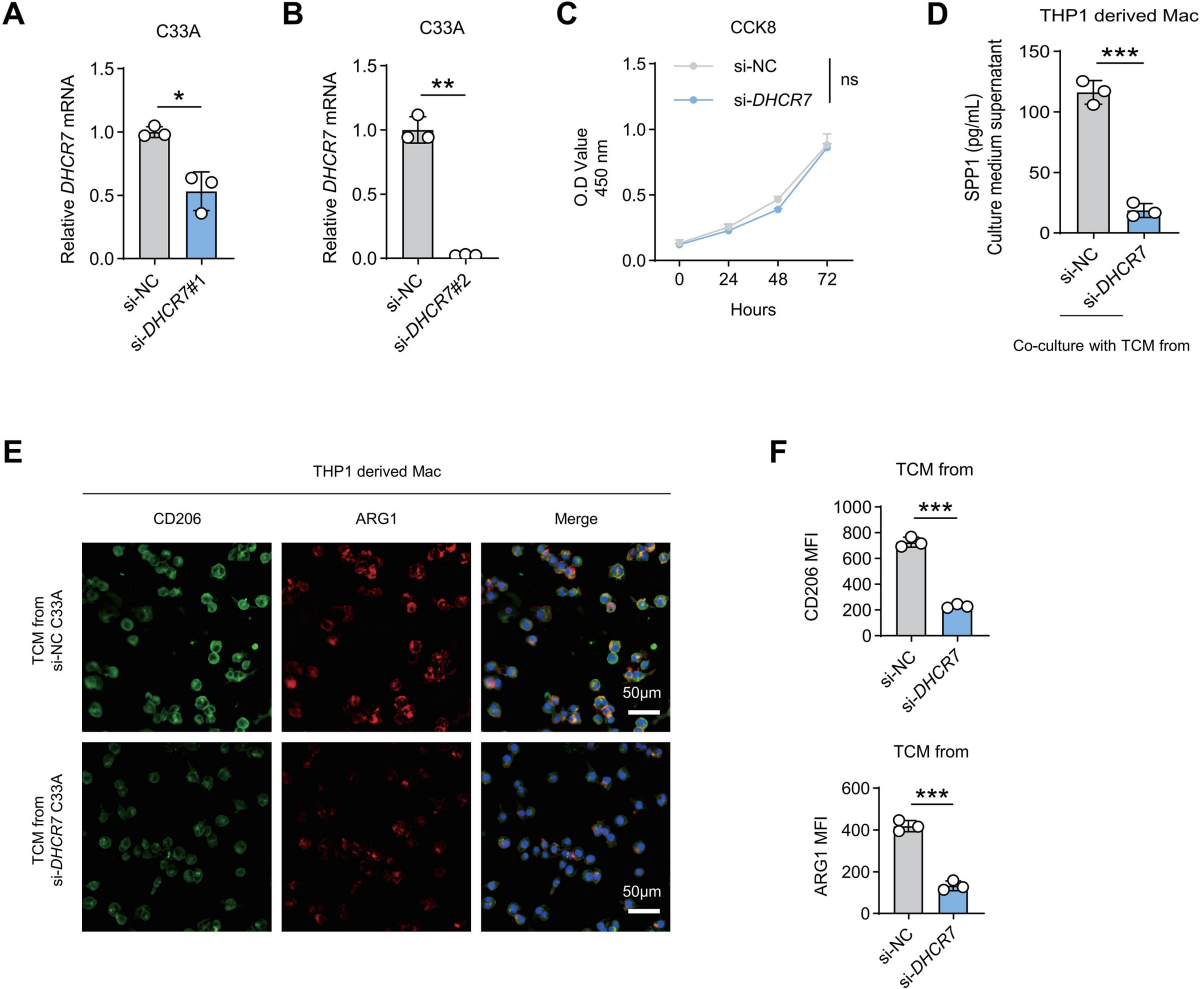
DHCR7 knockdown reverses macrophage M2 polarization induced by C33A cells. **(A, B)** qPCR analysis confirming *DHCR7* knockdown efficiency in C33A cells transfected with si-NC, si-DHCR7#1, or si-DHCR7#2. **(C)** CCK-8 assay showing cell viability of C33A cells following DHCR7 knockdown. **(D)** ELISA analysis of SPP1 secretion by THP-1–derived macrophages cultured with conditioned medium from control or DHCR7-silenced C33A cells. **(E, F)** Immunofluorescence staining of CD206 and ARG1 expression in macrophages cultured with conditioned medium from control or DHCR7-silenced C33A cells. Scale bar, 50 μm. * p < 0.05, ** p < 0.01, *** p < 0.001.

To determine whether DHCR7 is causally involved in macrophage modulation, DHCR7 was silenced in C33A cells using two independent siRNAs. qPCR analysis confirmed that both si-DHCR7#1 and si-DHCR7#2 effectively reduced DHCR7 expression compared with the negative control, with si-DHCR7#2 exhibiting superior knockdown efficiency (**Figures 7A, B**); therefore, si-DHCR7#2 was used in subsequent experiments. CCK-8 assays showed that DHCR7 knockdown had no significant effect on C33A cell viability (**Figure 7C**), excluding confounding effects related to cell proliferation. Conditioned media derived from DHCR7-silenced or control C33A cells were then applied to THP-1–derived macrophages. ELISA analysis revealed that macrophages treated with si-DHCR7-conditioned medium secreted significantly less SPP1 compared with those treated with control medium (**Figure 7D**). Consistently, immunofluorescence staining showed that conditioned medium from DHCR7-silenced C33A cells failed to induce CD206 and ARG1 expression in macrophages (**Figures 7E, F**). These results indicate that elevated DHCR7 expression in HPV-negative ADC cells is required for inducing macrophage immunosuppressive polarization and SPP1 production.

### SPP1 promotes DHCR7 expression and metabolic homeostasis in C33A cells via CD44

**Figure 8:**
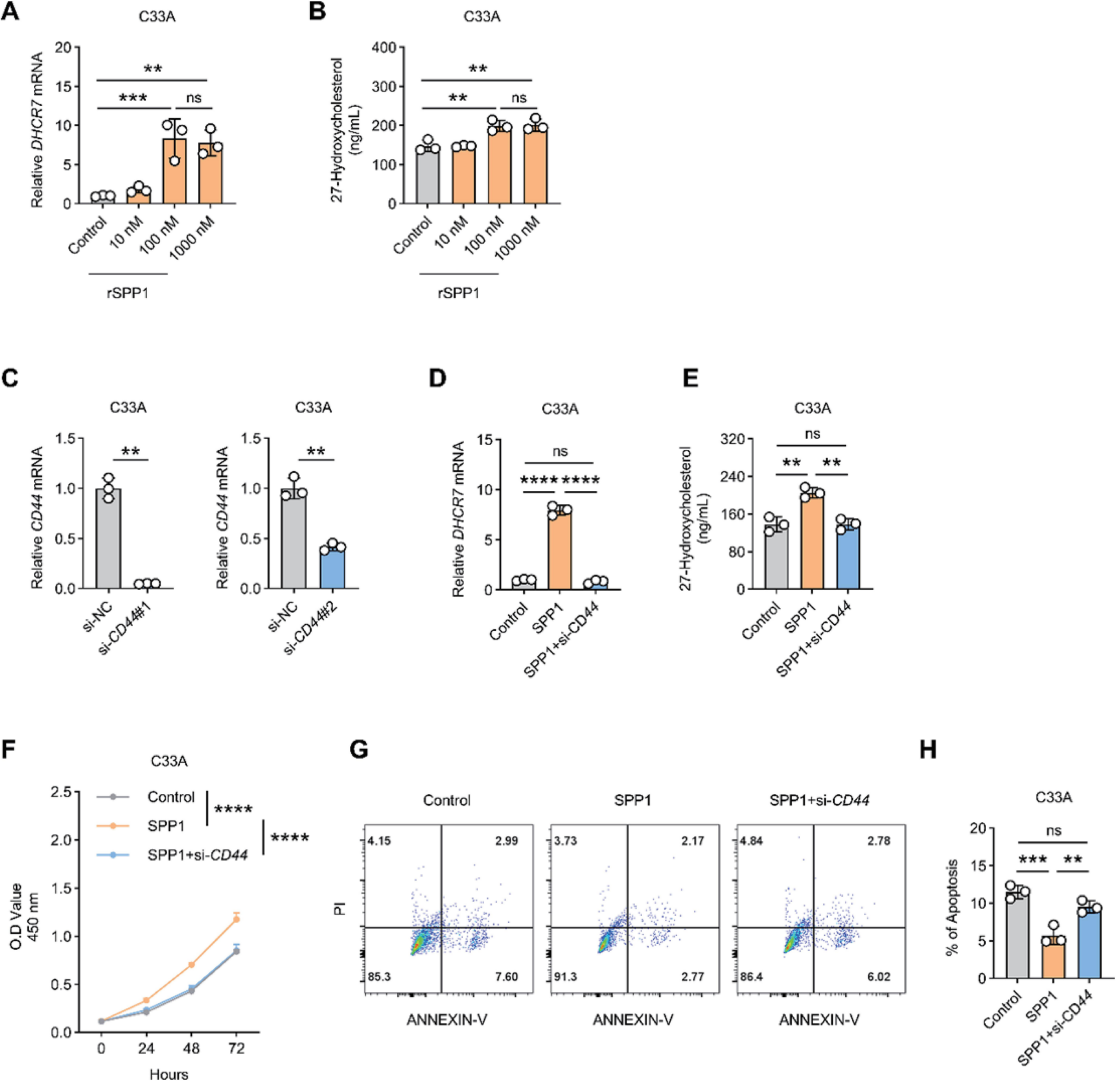
SPP1 promotes DHCR7 expression, 27-HC accumulation, and cell survival via CD44. **(A)** qPCR analysis of *DHCR7* expression in C33A cells treated with increasing concentrations of recombinant SPP1. **(B)** Quantification of intracellular 27-HC levels following rSPP1 treatment. **(C)** qPCR validation of *CD44* knockdown efficiency in C33A cells. **(D, E)** qPCR analysis of *DHCR7* expression and quantification of 27-HC levels in rSPP1-treated C33A cells following CD44 knockdown. **(F)** CCK-8 assay showing cell viability of C33A cells under indicated treatments. **(G, H)** Flow cytometric analysis of apoptosis in C33A cells following rSPP1 treatment with or without CD44 knockdown. Data are presented as mean ± SD. ns, not significant. ** p < 0.01, *** p < 0.001, **** p < 0.0001.

To explore the downstream effects of SPP1, we investigated whether SPP1 regulates tumor cell metabolism through the CD44 receptor. Treatment of C33A cells with recombinant SPP1 (rSPP1) resulted in a dose-dependent increase in DHCR7 mRNA expression (**Figure 8A**) and intracellular 27-HC levels (**Figure 8B**). Knockdown efficiency of CD44 was validated using two independent siRNAs, with si-CD44#1 showing stronger inhibitory effects (**Figure 8C**). Importantly, CD44 silencing markedly attenuated rSPP1-induced upregulation of DHCR7 (**Figure 8D**) and accumulation of 27-HC (**Figure 8E**). Functional assays further demonstrated that rSPP1 treatment enhanced C33A cell viability, promoted proliferation, and reduced apoptosis, whereas these effects were completely abolished following CD44 knockdown (**Figures 8F–H**). Collectively, these findings establish a positive regulatory loop involving the SPP1–CD44–DHCR7–27-HC axis, which reinforces metabolic reprogramming and supports tumor cell survival in HPV-negative ADC.

### Conditioned medium from DHCR7-silenced C33A cells alleviates CD8^+^ T cell exhaustion

**Figure 9:**
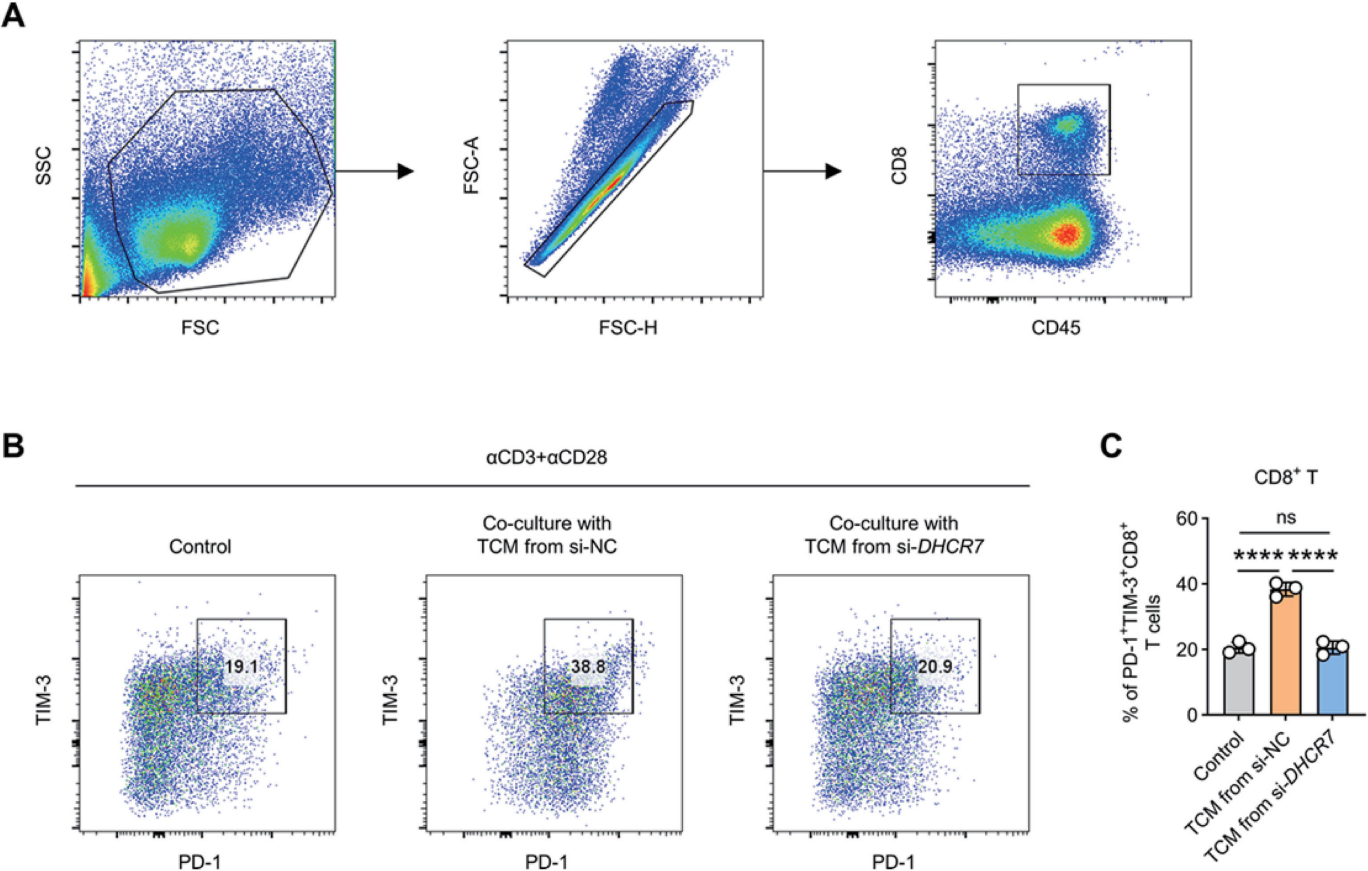
Conditioned medium from DHCR7-silenced C33A cells alleviates CD8^+^ T cell exhaustion. **(A)** Gating strategy for flow cytometric analysis of CD8^+^ T cells. **(B, C)** Representative flow cytometry plots and quantitative analysis of PD-1^+^TIM-3^+^ cells among CD8^+^ T cells cultured with conditioned medium from control or DHCR7-silenced C33A cells. Data are presented as mean ± SD. ns, not significant, **** p < 0.0001.

Finally, we examined the impact of DHCR7-mediated metabolic reprogramming on T cell function. Activated CD8^+^ T cells were cultured with conditioned medium from control or DHCR7-silenced C33A cells. Flow cytometric analysis revealed that CD8^+^ T cells exposed to conditioned medium from si-DHCR7-treated C33A cells exhibited significantly reduced expression of exhaustion markers PD-1 and TIM-3 compared with those treated with control conditioned medium (**Figures 9A–C**). These results indicate that HPV-negative ADC cells can drive CD8^+^ T cell exhaustion through DHCR7-dependent metabolic mechanisms, thereby contributing to suppression of antitumor immune responses.

## Discussion

In this study, we integrated single-cell transcriptomic analyses with functional experiments to systematically dissect the cellular, immunological, and metabolic heterogeneity of cervical adenocarcinoma stratified by HPV status. Our findings demonstrate that HPV-negative and HPV-positive cervical adenocarcinomas are not merely etiologically distinct but are characterized by fundamentally different tumor microenvironmental architectures, immune regulatory programs, and metabolic dependencies. In particular, we identify a previously unrecognized DHCR7–27-hydroxycholesterol–SPP1 axis that links malignant cell–intrinsic cholesterol metabolism to macrophage-mediated immunosuppression and CD8^+^ T cell exhaustion in HPV-negative cervical adenocarcinoma.

Single-cell profiling revealed marked differences in immune cell composition between HPV-negative and HPV-positive tumors. HPV-negative adenocarcinomas exhibited increased immune cell infiltration, particularly lymphocytes, yet this infiltration was accompanied by profound immune dysfunction, including enrichment of exhausted CD8^+^ T cells and immunosuppressive macrophage populations. This immune phenotype is consistent with a chronically inflamed but immunologically ineffective tumor microenvironment, which has been described in multiple cancer types associated with resistance to immunotherapy (23–25). In contrast, HPV-positive adenocarcinomas displayed relative immune exclusion, likely reflecting immune evasion mechanisms driven by viral oncogenes, including suppression of antigen presentation and disruption of interferon signaling (26, 27).

Our results further highlight macrophage heterogeneity as a central determinant of immune regulation in cervical adenocarcinoma. We identified a selective enrichment of SPP1^+^ macrophages in HPV-negative tumors, a subset that has been increasingly recognized as a key driver of immune suppression, tumor invasion, and poor prognosis across solid malignancies (28, 29). Pseudotime trajectory analysis revealed that Macro_C1QA cells in HPV-negative adenocarcinoma undergo a distinct evolutionary program characterized by progressive upregulation of SPP1, MIF, and stress-response genes, suggesting sustained functional reprogramming rather than transient activation. These findings indicate that macrophage-mediated immune suppression is not merely a secondary consequence of tumor growth but represents an actively maintained state in HPV-negative disease.

Beyond immune cell composition, our study identifies tumor metabolic reprogramming as a critical upstream driver of immune dysfunction. Pathway analyses revealed that steroid and cholesterol biosynthesis pathways were selectively upregulated in malignant epithelial cells from HPV-negative adenocarcinoma, with desmosterol emerging as a key enriched intermediate. Metabolic reprogramming is now recognized as a hallmark of cancer, enabling tumor cells not only to sustain proliferation but also to modulate the immune microenvironment (30, 31). In particular, cholesterol metabolites and oxysterols have been shown to function as bioactive signaling molecules that regulate immune cell migration, polarization, and effector function (32, 33).

Moreover, we demonstrate that DHCR7, a key enzyme in cholesterol biosynthesis, is highly expressed in HPV-negative cervical adenocarcinoma cells and drives accumulation of the oxysterol 27-HC. Prior studies in breast, lung, and colorectal cancer have shown that tumor-derived oxysterols promote recruitment of immunosuppressive myeloid cells, impair dendritic cell trafficking, and inhibit cytotoxic T cell responses (34–36). Our findings extend these observations to cervical adenocarcinoma and establish a direct causal link between DHCR7-dependent metabolic rewiring and macrophage immunosuppressive polarization.

Importantly, our data reveal a bidirectional feedback loop between malignant cells and macrophages. We show that SPP1 secreted by macrophages acts on tumor cells through the CD44 receptor, further enhancing DHCR7 expression and 27-HC production, thereby reinforcing metabolic reprogramming and tumor cell survival. Such self-sustaining metabolic–immune circuits have recently been proposed as key mechanisms underlying persistent immune suppression and therapeutic resistance in advanced cancers (37, 38). This SPP1–CD44–DHCR7–27-HC loop provides a mechanistic explanation for why HPV-negative tumors exhibit extensive immune infiltration yet remain refractory to effective antitumor immunity.

The functional consequences of this metabolic–immune axis are underscored by our T cell assays. Conditioned medium from DHCR7-high tumor cells promoted CD8^+^ T cell exhaustion, whereas DHCR7 knockdown partially restored T cell function, as evidenced by reduced expression of exhaustion markers. These findings align with accumulating evidence that metabolite-mediated signaling within the tumor microenvironment plays a decisive role in shaping T cell fate and limiting responses to immune checkpoint blockade (39, 40). Clinically, HPV-negative cervical cancers have demonstrated poor responses to PD-1/PD-L1–based immunotherapy (41), and our data suggest that targeting cholesterol metabolism may represent a rational strategy to overcome immune resistance in this subgroup.

Taken together, our study supports a model in which HPV-negative cervical adenocarcinoma is driven by tumor-intrinsic metabolic reprogramming that actively sculpts an immunosuppressive microenvironment through macrophage polarization and CD8^+^ T cell dysfunction. In contrast, HPV-positive tumors appear to rely more heavily on immune exclusion mechanisms linked to viral oncogenesis. These findings underscore the importance of HPV-based stratification when designing immunotherapeutic or metabolism-targeted interventions for cervical adenocarcinoma.

Despite providing a comprehensive characterization of the metabolic–immune regulatory axis centered on DHCR7–27-hydroxycholesterol–SPP1 in HPV-negative cervical adenocarcinoma, several limitations of this study should be acknowledged. First, the number of HPV-negative adenocarcinoma samples included in the single-cell transcriptomic analysis was relatively limited, which may not fully capture the intrinsic heterogeneity of this tumor subtype. Validation in larger, independent cohorts will be necessary to confirm the generalizability of the identified cellular states and signaling programs. Second, although our *in vitro* experiments using human cell lines and primary immune cells established causal links between tumor metabolic reprogramming and immune suppression, the absence of *in vivo* models precludes direct assessment of the DHCR7-driven metabolic–immune axis within an intact tumor microenvironment. Future studies employing orthotopic mouse models or patient-derived organoid–immune cell co-culture systems would be valuable to validate these findings in a more physiologically relevant context. Finally, the mechanisms underlying immune exclusion in HPV-positive cervical adenocarcinoma were not explored in depth in this study, and whether HPV-specific viral oncogenes interact with metabolic or stromal pathways to shape the tumor microenvironment warrants further investigation. Addressing these limitations in future studies will not only refine our mechanistic understanding but also facilitate the development of metabolism-based therapeutic strategies tailored to HPV-negative cervical adenocarcinoma.

## Conclusion

In summary, this study demonstrates that HPV-negative cervical adenocarcinoma is driven by a distinct metabolic–immune program characterized by DHCR7-mediated cholesterol reprogramming, immunosuppressive macrophage polarization, and T cell exhaustion. By contrast, HPV-positive ADC appears to rely more heavily on immune exclusion mechanisms associated with viral oncogenesis. These findings not only deepen our understanding of cervical ADC heterogeneity but also highlight DHCR7 and cholesterol metabolism as potential therapeutic vulnerabilities, particularly in HPV-negative disease. Future studies evaluating pharmacologic inhibition of cholesterol biosynthesis or SPP1–CD44 signaling in combination with immunotherapy may open new avenues for precision treatment of this aggressive cancer subtype.

## Data Availability

The original contributions presented in the study are included in the article/**Supplementary Material**. Further inquiries can be directed to the corresponding author.

## Supplementary material

Supplementary Figure 1 Heterogeneity of cancer associated fibroblasts. **(A)**UMAP visualization of CAF subpopulations. **(B)**Dot plot showing marker gene expression used to annotate CAF subtypes. **(C)** Relative proportions of CAF subpopulations across different sample groups.

Supplementary Figure 2 Copy number variation analysis of epithelial cells in cervical adenocarcinoma. **(A)** Distribution of epithelial cells derived from different sample groups. **(B)** UMAP visualization of epithelial cells colored by inferred cell identity. **(C)** InferCNV analysis showing copy number variation profiles of epithelial cells across different sample groups.

Supplementary Figure 3 Functional heterogeneity of epithelial cells in cervical cancer. **(A)** UMAP visualization of stress-related gene signature scores in epithelial cells. **(B)** UMAP visualization of mesenchymal gene signature scores in epithelial cells. **(C)** Gene set variation analysis (GSVA) comparing pathway activities between normal and malignant epithelial cells. **(D)** KEGG pathway enrichment analysis of malignant epithelial cell subpopulations across different sample groups.
