# Supplementary figures and images for: Metabolic reprogramming of cholesterol biosynthesis drives macrophage-mediated immune suppression in HPV-negative cervical adenocarcinoma
Source: Front Immunol. 2026 May 13;17:1808107. doi: 10.3389/fimmu.2026.1808107 (PMC13212449; doi:10.3389/fimmu.2026.1808107)

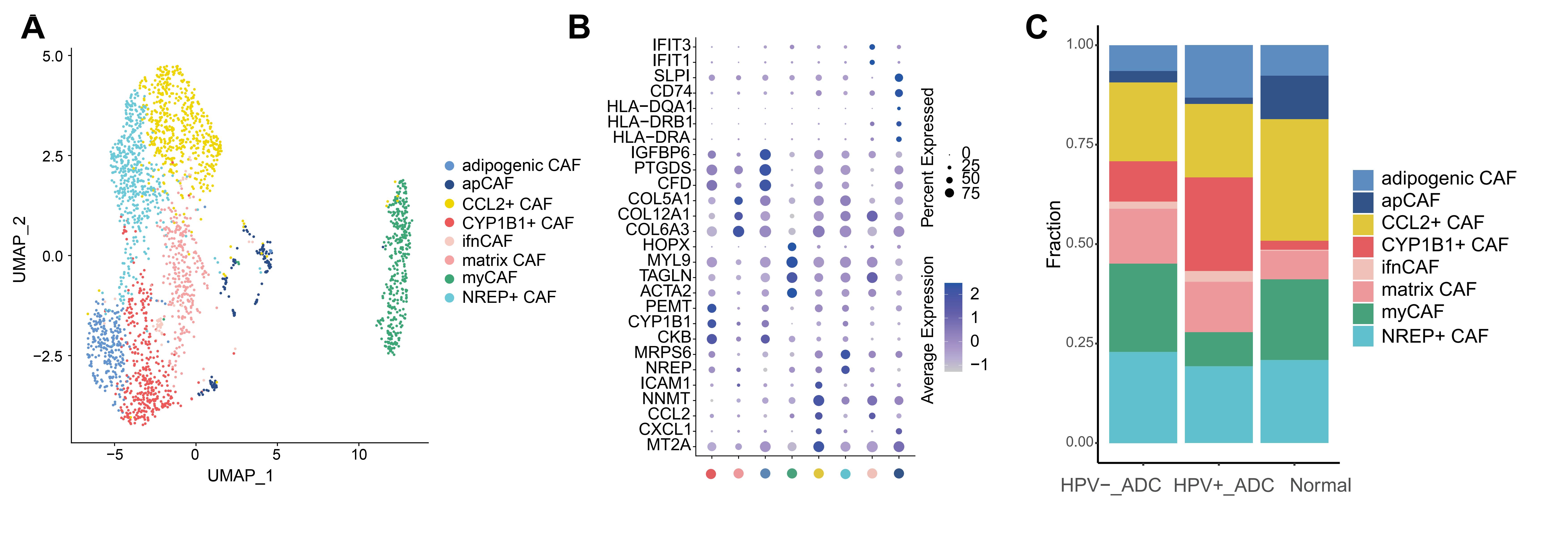

Supplement: Supplementary Figure 1 — Heterogeneity of cancer associated fibroblasts. (A)UMAP visualization of CAF subpopulations. (B)Dot plot showing marker gene expression used to annotate CAF subtypes. (C) Relative proportions of CAF subpopulations across different sample groups. [file Image1.jpeg]

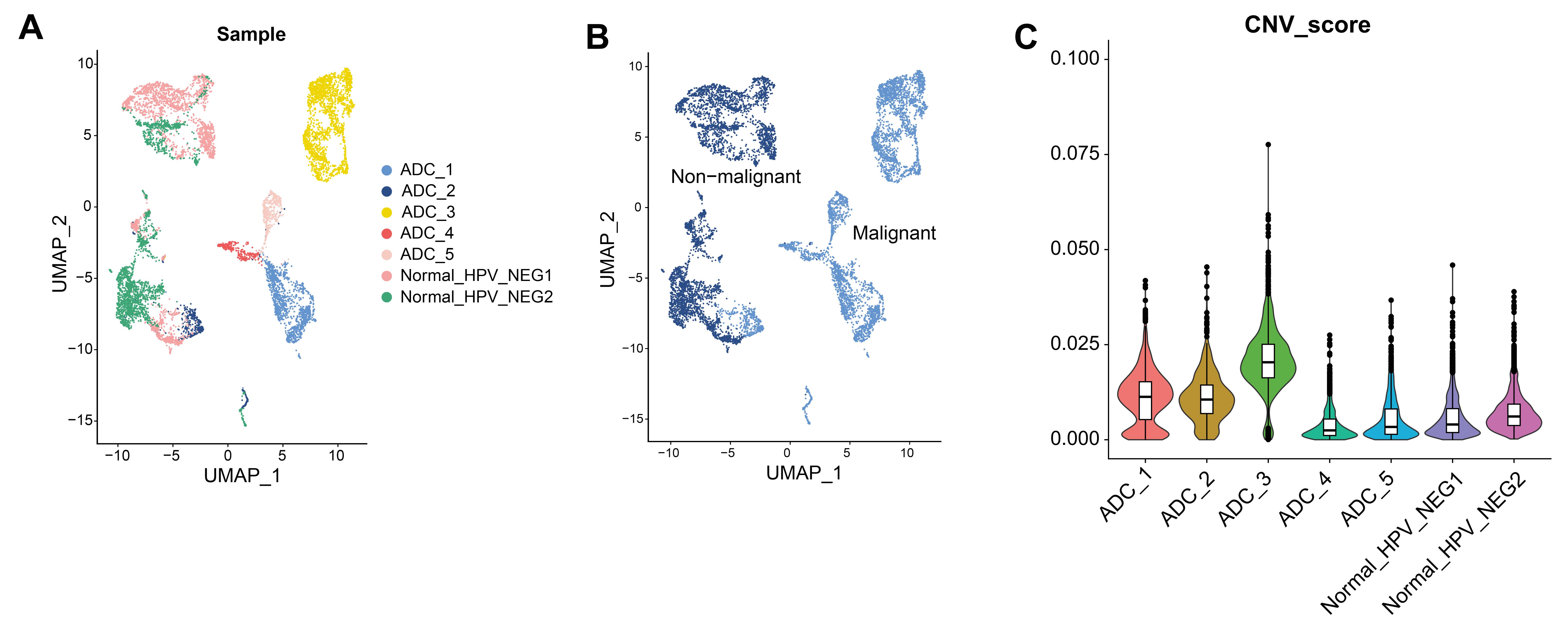

Supplement: Supplementary Figure 2 — Copy number variation analysis of epithelial cells in cervical adenocarcinoma. (A) Distribution of epithelial cells derived from different sample groups. (B) UMAP visualization of epithelial cells colored by inferred cell identity. (C) InferCNV analysis showing copy number variation profiles of epithelial cells across different sample groups. [file Image2.jpeg]

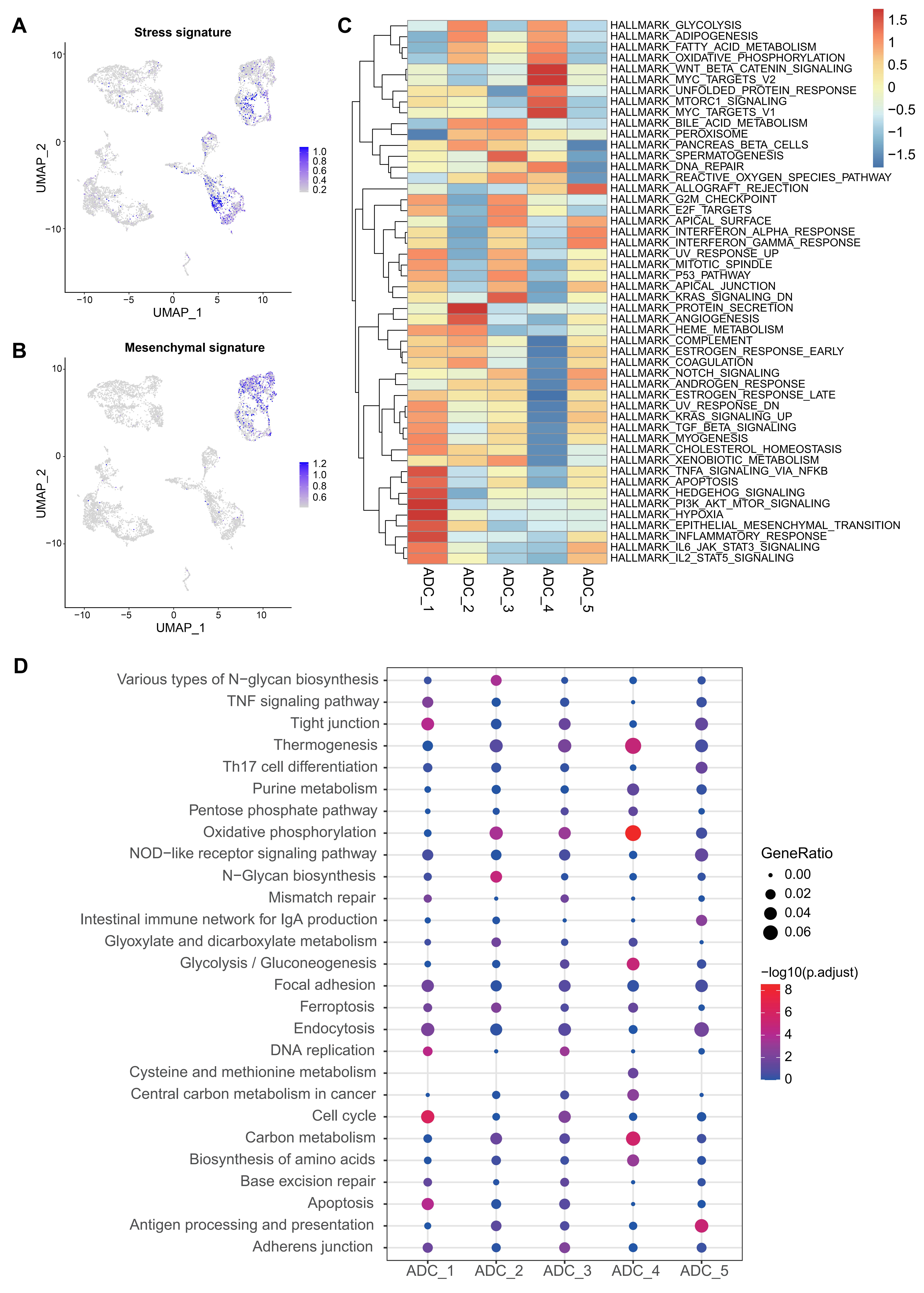

Supplement: Supplementary Figure 3 — Functional heterogeneity of epithelial cells in cervical cancer. (A) UMAP visualization of stress-related gene signature scores in epithelial cells. (B) UMAP visualization of mesenchymal gene signature scores in epithelial cells. (C) Gene set variation analysis (GSVA) comparing pathway activities between normal and malignant epithelial cells. (D) KEGG pathway enrichment analysis of malignant epithelial cell subpopulations across different sample groups. [file Image3.jpeg]
